# Multi-omics profiling reveal cells with novel oncogenic cluster, TRAP1^low^/CAMSAP3^low^, emerge more aggressive behavior and poor-prognosis in early-stage endometrial cancer

**DOI:** 10.1186/s12943-024-02039-2

**Published:** 2024-06-17

**Authors:** Xiaodan Mao, Xiaoyue Tang, Jingxuan Ye, Shuxia Xu, Yue Wang, Xianhua Liu, Qibin Wu, Xite Lin, Maotong Zhang, Jiangfeng Liu, Juntao Yang, Pengming Sun

**Affiliations:** 1 Laboratory of Gynecologic Oncology, College of Clinical Medicine for Obstetrics & Gynecology and Pediatrics, Fujian Maternity and Child Health Hospital, Fujian Medical University, Fuzhou, 350001 Fujian China; 2 Fujian Key Laboratory of Women and Children’s Critical Diseases Research, Fujian Maternity and Child Health Hospital (Fujian Women and Children’s Hospital), Fuzhou, 350001 Fujian China; 3 Fujian Clinical Research Center for Gynecological Oncology, Fujian Maternity and Child Health Hospital (Fujian Obstetrics and Gynecology Hospital), Fuzhou, 350001 Fujian China; 4 Pathology Department, Fujian Maternity and Child Health Hospital (Fujian Obstetrics and Gynecology Hospital), Fuzhou, 350001 Fujian China; 5 State Key Laboratory of Common Mechanism Research for Major Diseases, Department of Biochemistry and Molecular Biology, Institute of Basic Medical Sciences Chinese Academy of Medical Sciences, School of Basic Medicine Peking Union Medical College, Beijing, 100005 China

**Keywords:** TRAP1, CAMSAP3, TP53, Endometrial cancer, Tumor microenvironment, Proteomics, ScRNA-seq

## Abstract

The clinical heterogeneity of early-stage endometrial cancer (EC) is worthy of further study to identify high-quality prognostic markers and their potential role in aggressive tumor behavior. Mutation of TP53 was considered as an important primary triage in modified molecular typing for EC, it still cannot precisely predict the prognosis of EC. After proteomic analysis of cancer and para-cancerous tissues from 24 early-stage endometrioid EC patients with different survival outcomes, 13 differentially expressed proteins were screen out while 2 proteins enriched in p53 signaling pathway were further identified by single-cell transcriptome (scRNA-seq). Interestingly, tumor necrosis factor type-1 receptor-associated protein (TRAP1) and calmodulin-regulated spectrin-associated protein family member 3 (CAMSAP3) were found to be significantly downregulated in the specific cell cluster. Expectedly, the signature genes of TRAP1^low^/CAMSAP3^low^ cluster included classical oncogenes. Moreover, close cellular interactions were observed between myeloid cells and the TRAP1^low^/CAMSAP3^low^ cluster after systematically elucidating their relationship with tumor microenvironment (TME). The expression of TRAP1 and CAMSAP3 was verified by immunohistochemistry. Thus, a novel prediction model combining TRAP1, CAMSAP3 and TP53 was construct by multi-omics. Compared with the area under the curve, it demonstrated a significantly improvemrnt in the diagnostic efficacy in EC patients from TCGA bank. In conclusion, this work improved the current knowledge regarding the prognosis of early-stage EC through proteomics and scRNA-seq. These findings may lead to improvements in precise risk stratification of early-stage EC patients.

**Supplementary Information:**

The online version contains supplementary material available at 10.1186/s12943-024-02039-2.

## Introduction

Endometrial cancer (EC) ranks as the sixth most common malignant tumor globally [1]. Despite the favorable prognosis of EC, a subset of patients with early and low-grade lesions have a rapidly progressive course. The ProMisE and Trans-PORTEC, molecular classification systems modified from The Cancer Genome Atlas (TCGA) genomic subgroups have been feasibly used in clinical practice through immunohistochemistry (IHC) and are helpful for improving the diagnosis and providing better therapeutic strategies [2]. However, IHC revealed that approximately 48.2% of p53abn patients were missed [3]. The application of multi-omics techniques helps us to identify more meaningful biomarkers, but few studies have focused on prognostic proteins and the tumor microenvironment (TME) [4, 5]. Therefore, it is worthwhile to identify high-quality prognostic markers of early-stage EC and to explore the interactions of oncogenic subgroups with TME, improve the precision of p53abn detection and avoid missed diagnoses.

## Results

### Multi-omics identification of prognosis-associated proteins in early-stage EC and characterization of the TRAP1^low^/CAMSAP3^low^ cluster

**Fig. 1:**
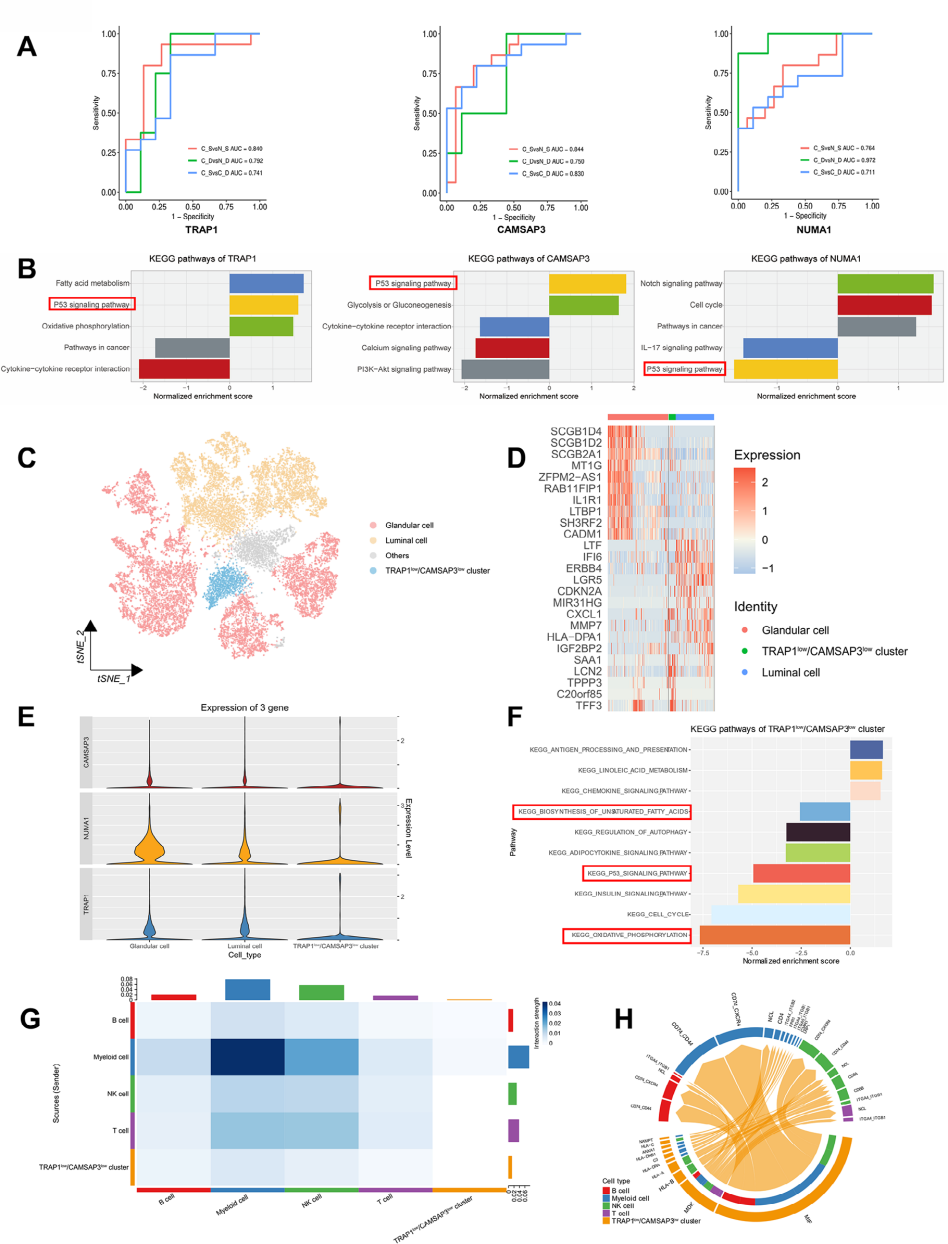
Multi-omics identification of prognostic proteins in early-stage EC and revealing characteristics of subtypes. **A** ROC curves of TRAP1, CAMSAP3 and NUMA1 in different groups. (C_D, Cancer tissues at death. N_D, Para-cancerous tissues in death. C_S, Cancer tissues in survival. N_S, Para-cancerous tissues in survival). **B** TRAP1, CAMSAP3 and NUMA1-related signaling pathways based on GSEA. **C** t-SNE plot showing clusters of epithelial types after reclustering. **D** Heatmap showing the top genes in different subpopulations after reclustering. **E** Violin plot showing the mean expression of *TRAP1*, *CAMSAP3* and *NUMA1* in the subgroups after regrouping. **F** GSEA analysis of the TRAP1^low^/CAMSAP3^low^ cluster. **G** CellChat plot of TRAP1^low^/CAMSAP3^low^ cluster and immune subpopulations. **H** Receptor-ligand pairs chords of TRAP1^low^/CAMSAP3^low^ cluster and immune cells

The brief process of this study was presented in Fig. S1. Clinical characteristics of stage I-II EC patients, including 15 survival and 9 dead patients, were listed in Table S1. The histopathological types of all patients were endometrioid endometrial cancer (EEC). Differentially expressed proteins (DEPs) of three categories, including tumor focal vs. para-cancerous tissues belonging to survival patients, tumor focal vs. para-cancerous tissues belonging to dead patients and tumor focal tissues of survival patients vs. dead patients, were identified (Fig. S2A). By comparing and analyzing the tumor focal tissues of survival and dead patients, the up-DEPs were significantly enriched in the oxidative phosphorylation. When comparing tumor focal and para-cancerous tissues belonging to survival patients, the up-DEPs were enriched in the synthesis of unsaturated fatty acids (Fig. S2B). 34 DEPs were performed LASSO analysis and 13 prognostic proteins were ultimately obtained through cross-matching aboved three categories: AK3, ATF2, NUMA1, CACHD1, ZMPSTE24, TRAP1, COG3, CAMSAP3, COX4I1, PGPEP1, UBL5, HADHA, and SNRPGP15 (Fig. S2C-E). The 13 candidate proteins showed good outcome prediction and diagnostic performance by ROC curves were screened (Fig. 1A and Fig. S2F). Five prognostic proteins, including TRAP1, CAMSAP3, NUMA1, UBL5, COX4I1 (Fig. 1B and Fig. S2G) were engaged in the p53 signaling pathway through GSEA analysis.

ScRNA-seq was carried out for 5 patients with stage IA EEC and 3 adjacent normal endometrial tissues from 3 patients (Table S2). After quality control and filtering, 79,641 high-quality cells were obtained, 5 main known cell types were clustered and identified: epithelial cells, stromal fibroblasts cells, endothelial cells, smooth muscle cells and immune cells (Fig. S3A-C). Through differential expression analysis via scRNA-seq, 3 differentially expressed genes (DEGs) consistent with the proteomic data: *NUMA1*, *TRAP1* and *CAMSAP3* (Fig. S3D), and their expression levels were showed in different clusters (Fig. S3E). The proportion of epithelial cells was significantly greater in tumors than in normal samples (Fig. S3F) and previous report has shown that EC originated from unciliated epithelial cells [4]. Therefore, the epithelial cells were firstly classified into 3 prime subclusters: luminal cells, glandular cells and ciliated epithelial cells (Fig. S3G-I). After excluding the ciliated epithelial cells, the epithelial cells were re-clustered, yielding 10 clusters. Then the expression levels of 3 genes were assessed in each cluster and the gene expressions of clusters 6 and 7 were lower than their median (*TRAP1*, median = 0.096, *CAMSAP3*, median = 0.040, Nuclear mitotic apparatus protein (*NUMA1)*, median = 0.138) (Fig. S4A and Table S3). By analyzing the unciliated epithelial cells in tumors and para-cancer samples, cluster 7 was predominantly present in normal cells, thus cluster 6 was the focus (Fig. S4B). Moreover, almost all other cells could be classified as glandular cells or luminal cells (Fig. 1C, D and Fig. S4C). The top genes of the cluster 6 included previously reported oncogenes, such as *LCN2, SAA1* and *TFF3*. *TRAP1, NUMA1* and *CAMSAP3* were highly expressed in glandular cells and luminal cells, but all lowly expressed in cluster 6 (Fig. 1E). Moreover, GSEA analysis of unciliated epithelial cells revealed that the p53 signaling pathway was downregulated in cluster 6 (Fig. 1F) and upregulated in glandular and luminal cells (Fig. S4D). These results were consistent with the GSEA of *TRAP1* and *CAMSAP3* derived proteomics, except *NUMA1*. Thus, we defined cluster 6 as the TRAP1^low^/CAMSAP3^low^ cluster, and then evaluated its basic features in the TME.

Analysis of intercellular interactions with CellChat revealed that epithelial cells interacted most closely with immune cells (Fig. S5A). To identify which immune subclusters interacted intimately with the TRAP1^low^/CAMSAP3^low^ cluster, the immune subclusters were assigned cell identity, including T cells, B cells, NK cells, and myeloid cells (Fig. S5B-D). The interaction intensity between the TRAP1^low^/CAMSAP3^low^ cluster and myeloid cells was the greatest (Fig. 1G). The top ligand-receptors included *MIF- CD74/CD44*, *MIF-CD74/CXCR4, MDK-NCL* etc. (Fig. 1H). Subsequently, the myeloid cells were divided into three clusters: dendritic cells (DCs), macrophages and monocytes (Fig. S5E-G). The interaction between the TRAP1^low^/CAMSAP3^low^ cluster and macrophages was the greatest (Fig. S5H), and the top receptor-ligand pairs almost overlapped with those in myeloid cells (Fig. S5I). The results indicated that macrophages are likely to be the main cells that interact with the TRAP1^low^/CAMSAP3^low^ cluster of myeloid cells, and the major receptor-ligands include *MIF-CD74/CXCL4, MIF-CD74/CD44*.

### Validation of prognostic proteins and their ability to predict mortality risk in EC patients

**Fig. 2:**
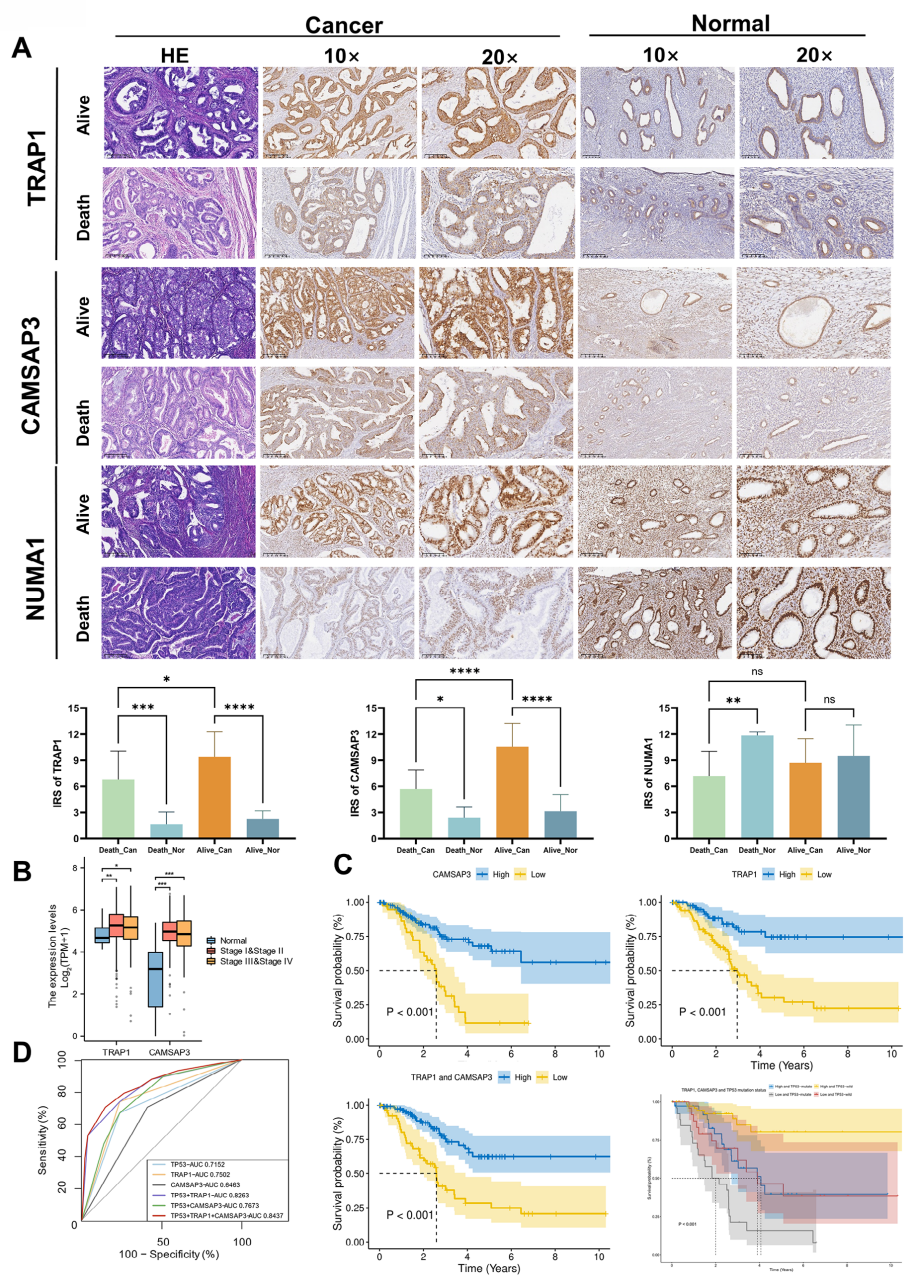
Verification of prognostic proteins in clinical. **A** Representative images of immunohistochemical staining for TRAP1, CAMSAP3 and NUMA1 in cancer tissues and normal tissues of EC patients with different prognosis and statistical box plot of prognostic protein expression in different groups. (*p < 0.05; **p < 0.01; ***p < 0.001.) **B** The expression of *TRAP1* and *CAMSAP3* was validated in early (Stage I-II, *n* = 386), advanced (Stage III-IV, *n* = 153) stages of EC as well as in normal patients (*n* = 35) through the TCGA database. (Kruskal-Wallis test, p-values are noted). The expression level of each gene was visualized by log_2_(TPM + 1). Box plots showed the median (center), 25–75 percentile (box), and lower whisker = smallest observation greater than or equal to lower hinge − 1.5 * IQR (interquartile range); upper whisker = largest observation less than or equal to upper hinge + 1.5 * IQR. **C** Combination with *TRAP1*, *CAMSAP3* and *TP53* mutation status predicted a disease outcome across TCGA EC cohorts. The median was set as the cut-off value to stratify EC patients into low-expression and high-expression groups. Survival curves were visualized by Kaplan–Meier method. **D** ROC curves of the predictive models were constructed by combining *TP53, TRAP1* and *CAMSAP3* from the TCGA database. The AUC for each model was shown

IHC was used to verify the expression of prognostic proteins in different samples. TRAP1 and CAMSAP3 expression was greater in the para-cancerous group than in the cancer group with different prognoses. The expression of TRAP1 and CAMSAP3 in tumor tissues of dead patients was lower than survival patients. However, the expression of NUMA1 did not change significantly in cancer and para-cancerous tissues of survival patients and in cancer tissues with different prognoses (Fig. 2A). The Youden index (Youden index = specificity + sensitivity − 1) was calculated according to the immune score of prognostic proteins, and the value corresponding to the maximum Youden index was selected as the cut-off value (TRAP1:5.5, CAMSAP3: 9.8, NUMA1: 6.95). The expression of TRAP1 and CAMSAP3 was consistent with the proteomic analysis, but the expression of NUMA1 did not match.

To further confirm the correlation between prognostic proteins expression and EC, and validate whether they can be used as independent prognostic proteins, we analyzed TCGA cohorts for external validation. The data were divided into early (stage I-II), advanced (stage III-IV) and adjacent tissues (normal). Differential expression comparison and survival analysis were performed (Fig. 2B). Then we selected the patients who died from EC (*n* = 57) and matched 110 surviving patients for analysis by propensity matching score (*N* = 167) in the TCGA database. The results showed that patients with low-expression scores which was calculated for the TRAP1 and CAMSAP3 signatures in endometrial lesions had significantly lower survival rates across the selected cohorts, which was consistent with our results (*p* < 0.05). The same results can be observed in patients with stage I-II EC and patients without TP53 mutation (Fig. S7 A, B). After combined analysis of TRAP1 and CAMSAP3 with TP53 mutation, it can be seen that the 5-year survival rate of patients decreased rapidly, suggesting the rapid progression of the disease (Fig. 2C). And the 5-year survival rate of patients significantly decreased, suggesting the worst prognosis of I-II EC patients (*p* < 0.05, Fig. S7B). A clinical predictive model was also constructed with the same data. The AUC for *TP53* combined with *TRAP1* and *CAMSAP3* was 0.84, which was significantly (*p* < 0.001) greater than that for *TP53* (AUC = 0.72) (Fig. 2D and Table S4). The results confirmed that the prognostic proteins we selected can be used for further research in the future.

## Discussion

Through multi-omics analysis, 2 prognostic proteins involved in p53 signaling pathway were chosen for further research. TRAP1, also known as heat shock protein 75, can help maintain stability of the mitochondrial internal environment [6]. In HeLa cells, TRAP1 bound to TP53-binding protein 1 and was involved in the post-transcriptional regulation of cellular primary cilia formation through interacting with syndesmos. Besides, the interaction regulated protein synthesis and contribute to the expression of metabolic gene changes in cancer cells. In addition, they identified TRAP1 can interact with MIF proteins [7]. These findings supported that EC originated from unciliated epithelium, and the TRAP1^low^/CAMSAP3^low^ cluster interacted with myeloid cells via MIF signaling again. Study has shown that when the expression level of TRAP1 decreased, the invasiveness of colon and renal clear cell carcinoma cells and the production of reactive oxygen species (ROS) increased [6]. High levels of ROS can promote can promote the polarization and production of tumor-associated macrophages and the accumulation of hypoxia-inducible factor-1α (HIF-1α) [8, 9]. CAMSAP3 can regulate microtubule stability and played a key role in the apical-to-basal polarity of microtubules in oriented epithelial cells [10]. Knockout of CAMSAP3 promoted invasion and malignant progression of lung cancer and the expression of HIF-1α [11]. The activation of HIF-1α was one of the main initiating factors of the interaction between macrophage and tumor cells [9]. In this study, low TRAP1 and CAMSAP3 expression was indicative of poor prognosis in EC patients. However, there was no significant difference in the expression trend of NUMA1 in EC samples with different prognoses, which is likely due to our small sample size. By analyzing prognostic proteins combined with scRNA-seq, an oncogenic epithelial cells was defined and observed with low *TRAP1* and *CAMSAP3* expression. And we speculated that TRAP1 and CAMSAP3 were associated with macrophages. After analyzing the top genes of this cluster, the top oncogenes *LCN2* and *SAA1* were found which were consistent with the findings of Chen’s work [4] and both of them were confirmed to contribute to the progression of malignant tumors. *TFF3*, a key oncogene that promoted colon cancer migration and invasion [12], was also reported in the top genes of this defined cells. Among the enriched pathways, p53 signaling pathway have been reported to be associated with EC progression [13]. TP53 is an important tumor repressor and one of the commonly mutated genes in human cancers. Gain-of-function *TP53* mutations or the dysregulation and deficiency of p53 signaling pathway facilitates tumorigenesis and even lead to poor patient prognosis. The *TP53* mutant p53-R248Q promoted EC migration and invasion by upregulating the transcription of the proteasome activator REGγ [14]. Consistently, p53 signaling pathway was downregulated in the TRAP1^low^/CAMSAP3^low^ cluster by scRNA-seq.

Furthermore, we found the TRAP1^low^/CAMSAP3^low^ cluster interacted closely with myeloid cells, especially macrophages through multiple ligand-receptors, including *MIF/CD74-CD44* and *MIF/CD74-CXCL4*. Previous studies have reported the interaction of fibroblasts and myeloid cells in colorectal cancer activated protumorigenic signaling pathways such as MIF/CD74 and promoted the aggressive phenotypes [15]. It is evident that myeloid cells played an indispensable role in TME.

The 2023 FIGO stage of EC now includes TP53 detection as a pivotal reference [2]. Although TP53 status accurately predicts patient outcomes, the sensitivity of the *TP53* mutation leaves much to be desired. To explore whether our screened prognostic proteins could improve the ability of *TP53* to predict poor prognosis in EC patients. After validating the prognostic proteins, we selected appropriate data from public databases to construct a predictive model. The AUCs of *TRAP1* and *CAMSAP3* were high in EC patients and significantly improved in combination with *TP53*, indicated that *TRAP1* and *CAMSAP3* can predict the risk of EC malignant progression. However, our study also has limitations. For example, when verifying prognostic proteins, the sample size was small. We will expand the sample size to further verify the prognostic proteins.

## Conclusion

In summary, TRAP1 and CAMSAP3 were closely related to the prognosis of early-stage EC patients and significantly improved the efficacy of TP53 detection via IHC. TRAP1^low^/CAMSAP3^low^ cluster was enriched and promoted carcinogenesis via p53 signaling pathway. Furthermore, the cluster is closely related to myeloid cells through receptor-ligand pairs interactions. Our datasets will be valuable resources for further exploration of the molecular mechanisms underlying malignant transformation in early-stage EC. This research provides a theoretical basis for early prognostic factors for triaging EC patients.

### Electronic supplementary material

Below is the link to the electronic supplementary material.

**Supplementary Material 1: Fig. S1 Research flow chart**. This research began by comparing proteins in early-stage endometrial cancer (EC) patients with varying outcomes using proteomics. LASSO regression was used to identify 13 key proteins. After a thorough literature review and protein function prediction and classification, we identified 5 crucial proteins involved in the p53 signaling pathway. The expression of these proteins was confirmed by single-cell transcriptome (scRNA-seq). The interactions between cellular subpopulations and the intercellular communication between the TRAP1^low^/CAMSAP3^low^ cluster and the tumor microenvironment (TME) have received particular attention. Finally, the prognostic proteins were validated by immunohistochemistry (IHC) and data from the TCGA database. Based on this, a TP53-based model to predict outcomes for early-stage EC was created and optimized. This figure was created with BioRender.com

**Supplementary Material 2: Fig. S2 Screening Prognosis-Related Proteins with Proteomics**. **A** Volcano plots of tumor focal vs para-cancerous tissues belonging to survival patients, tumor focal vs para-cancerous tissues belonging to dead patients and tumor focal tissues of survival patients vs dead patients. **B** KEGG pathway enrichment analysis of differential proteins. **C** Venn diagram of the intersection of three groups of differential proteins. **D** LASSO regression analysis for differential proteins yielded prognosis-associated proteins and their expression in different subgroups. **E** The changes of prognosis-associated proteins expression in different subgroups. F ROC curves of prognosis-associated proteins in different groups. **G** GSEA analysis of COX4I1 and UBL5-related signaling pathways. (*p<0.05; **p<0.01; ***p<0.001. C_D, Cancer tissues in death. N_D, Para-cancerous tissues in death. C_S, Cancer tissues in survival. N_S, Para-cancerous tissues in survival)

**Supplementary Material 3: Fig. S3 The derived cellular subpopulations of prognostic proteins were analyzed at the transcriptomic level**. **A** t-SNE plots of cells from five patients (8 samples). Colors represented cell types. Cells were clustered into 5 cell types based on biological annotation. Each dot represents a single cell. **B** t-SNE plots of canonical markers for major cell types. **C** The expression levels of canonical marker genes for the above 5 cell types. Circle size represents the percentage of cells that expressed the gene, and colors represented the average expression value within a cluster. **D** The Venn plot showed the intersection between the prognostic proteins involved in the p53 signaling pathway identified by proteomics in tumor focal and para-cancerous tissues of survival patients and the differential genes of scRNA-seq. **E** Bar plot showing the expression of 3 genes in different cell types. **F** Bar chart showing the relative proportion of major cell types in each sample. **G** t-SNE plot showing clusters of epithelial. **H** Top genes in different cell populations of epithelial cells. **I** t-SNE plots of canonical markers for epithelial cells

**Supplementary Material 4: Fig. S4 Expression of target protein in subpopulations**. **A** Violin plot showed average gene expression of different clusters across all unciliated epithelial cells. **B** tSNE plot of the distribution of epithelial cells other than ciliated epithelial cells in tumor and normal samples. **C** t-SNE plots of canonical markers for epithelial subtypes. **D** GSEA analysis of glandular cells and luminal cells

**Supplementary Material 5: Fig. S5 Myeloid cells, especially macrophages interacted closely with TRAP1**^**low**^**/CAMSAP3**^**low**^ **cluster**. **A** CellChat plot of intercellular communication between different cell types. **B** t-NSE plot of immune cells showed its major cell types. **C** Top genes in different cell subpopulations of immune cells. **D** Expression of cellular marker for each subpopulation of immune cells. **E** t-NSE diagram of myeloid cells. **F** Expression of cellular marker of different subpopulation in myeloid cells. **G** Top genes in different cell populations of myeloid cells. **H** CellChat plot of TRAP1^low^/CAMSAP3^low^ cluster and myeloid cells. **I** Bubble chart showing the expression of receptor-ligand pairs in TRAP1^low^/CAMSAP3^low^ cluster and myeloid cells

**Supplementary Material 6: Fig. S6 Survival curves of combined TRAP1, CAMSAP3 and TP53 mutation status across TCGA cohorts**. **A** Combination with TRAP1 and CAMSAP3 predicted a disease outcome in EC patients without TP53 mutation. **B** Combination with TRAP1, CAMSAP3 and TP53 mutation status predicted a disease outcome in 106 stage I-II EC patients. Survival curves were visualized by Kaplan– Meier method. The endpoint was 10 years. The median was set as the cut-off value to stratify EC patients into low-expression and high-expression groups

**Supplementary Material 7: Table S1** Clinical characteristics of early-stage endometrioid endometrial cancer patients performed by proteomics. **Table S2** Clinical characteristics of the IA stage (FIGO 2009) patients performed by scRNA-seq. **Table S3** The mean expression levels (TPM) of prognostic genes in different clusters of unciliated epithelium. **Table S4** The efficiency of different models on survival outcomes in EC patients from TCGA bank (N = 167). **Supplementary methods and materials. Supplementary reference**

## Data Availability

The datasets used and/or analyzed during the current study are available from the corresponding author on reasonable request. No new algorithms were developed for this manuscript.

## Abbreviations

EC: Endometrial cancer
scRNA-seq: Single-cell transcriptome
TRAP1: Tumor necrosis factor type-1 receptor-associated protein
CAMSAP3: Calmodulin-regulated spectrin-associated protein family member 3
TME: Tumor microenvironment
TCGA: The Cancer Genome Atlas
FIGO: Federation of Gynecology and Obstetrics
EEC: Endometrioid endometrial cancer
DEPs: Differentially expressed proteins
DEGs: Differentially expressed geness
NUMA1: Nuclear mitotic apparatus protein
DCs: Dendritic cells
PSM: Propensity score matching
EMT: Epithelial-mesenchymal transition
FFPE: Formalin-fixed, paraffin-embedded
KEGG: Kyoto Encyclopedia of Genes and Genomes
GSEA: Gene set enrichment analysis
t-SNE: t-distributed stochastic neighbor embedding
IHC: Immunohistochemistry
