# Supplementary figures and images for: Multi-omics profiling reveal cells with novel oncogenic cluster, TRAP1low/CAMSAP3low, emerge more aggressive behavior and poor-prognosis in early-stage endometrial cancer
Source: Mol Cancer. 2024 Jun 17;23:127. doi: 10.1186/s12943-024-02039-2 (PMC11181528; doi:10.1186/s12943-024-02039-2)

# Stage I-II Endometrioid endometrial cancer

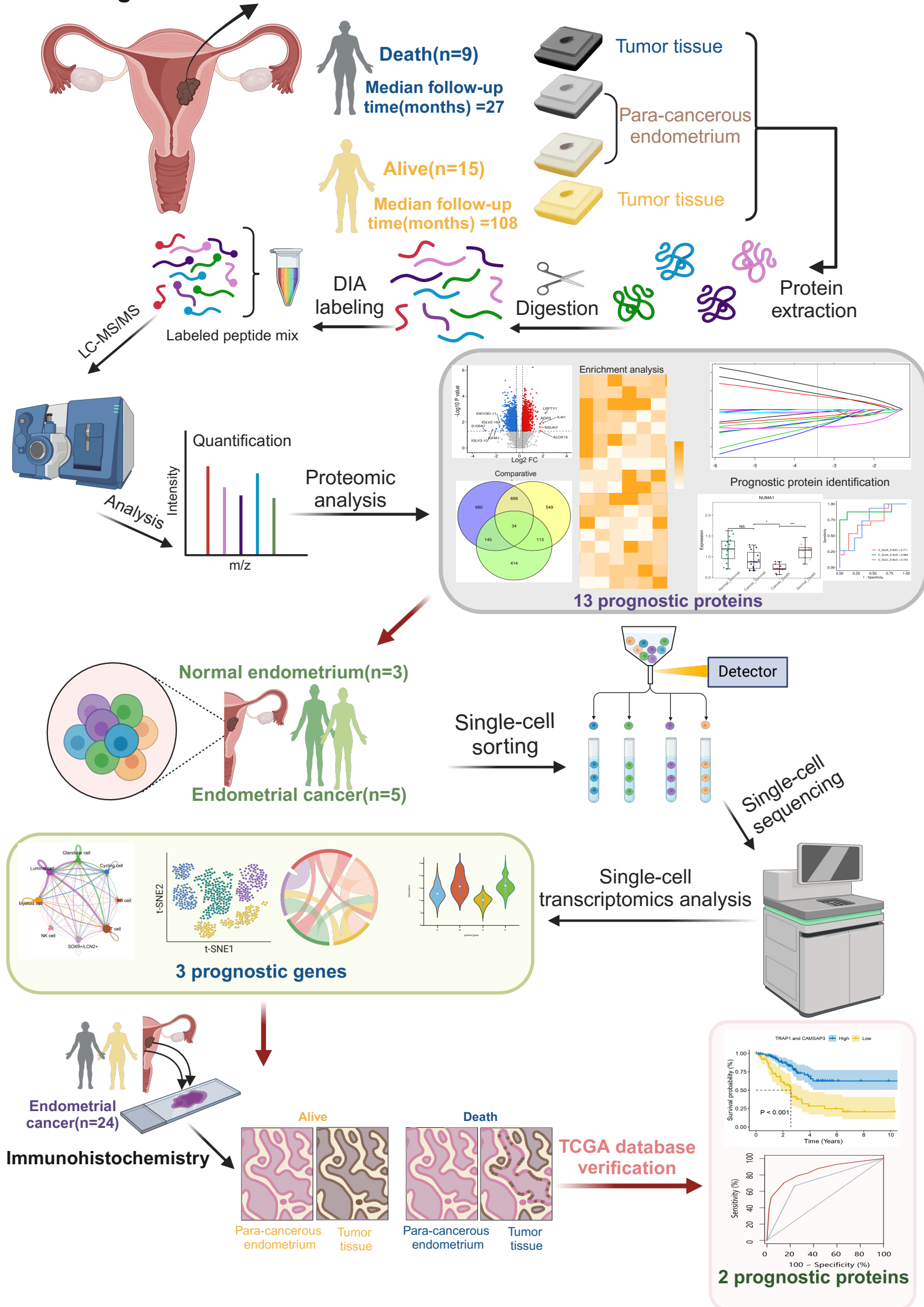

Supplement: Supplementary file 1 — Supplementary Material 1: Fig. S1 Research flow chart. This research began by comparing proteins in early-stage endometrial cancer (EC) patients with varying outcomes using proteomics. LASSO regression was used to identify 13 key proteins. After a thorough literature review and protein function prediction and classification, we identified 5 crucial proteins involved in the p53 signaling pathway. The expression of these proteins was confirmed by single-cell transcriptome (scRNA-seq). The interactions between cellular subpopulations and the intercellular communication between the TRAP1low/CAMSAP3low cluster and the tumor microenvironment (TME) have received particular attention. Finally, the prognostic proteins were validated by immunohistochemistry (IHC) and data from the TCGA database. Based on this, a TP53-based model to predict outcomes for early-stage EC was created and optimized. This figure was created with BioRender.com [file 12943_2024_2039_MOESM1_ESM.pdf]

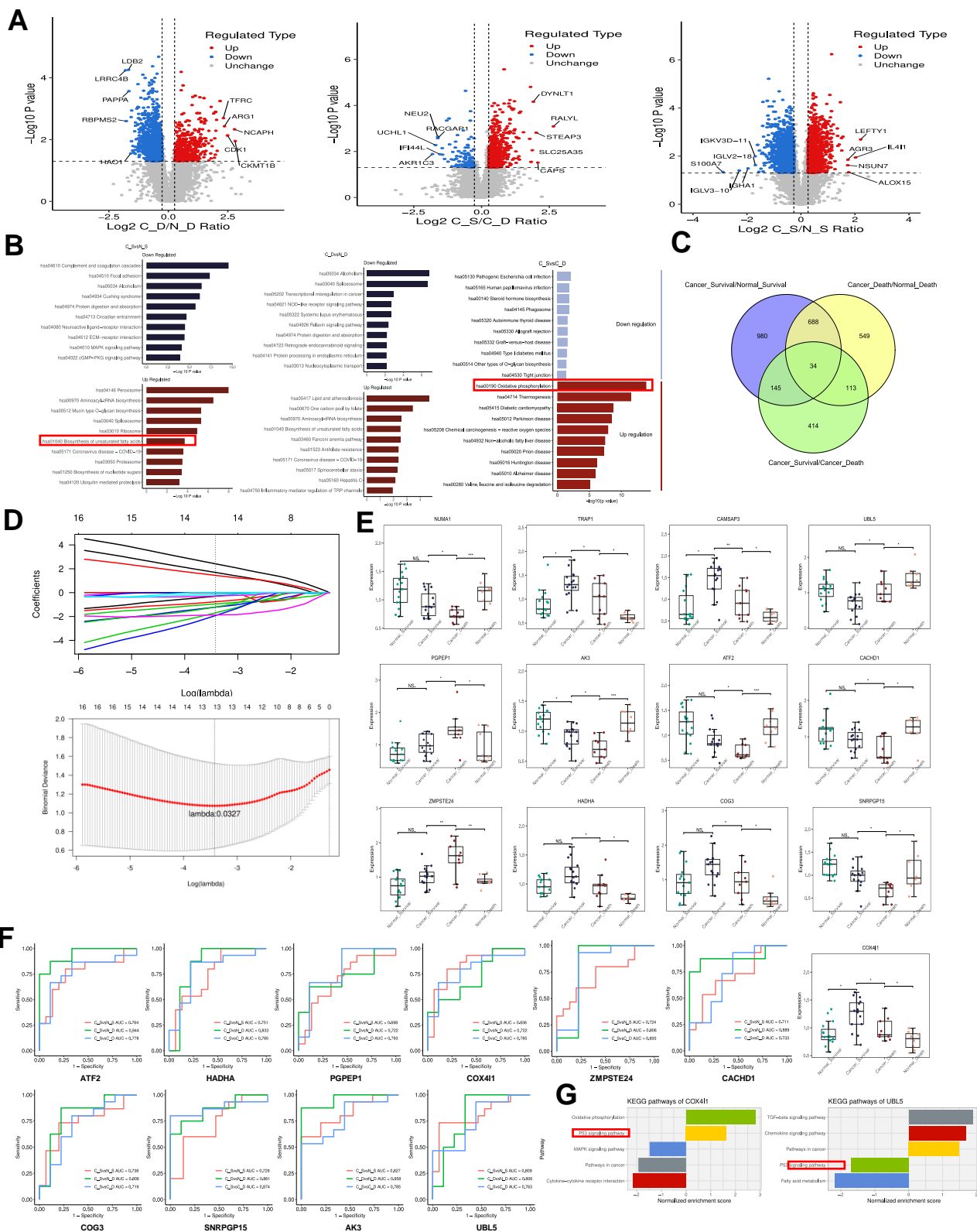

Supplement: Supplementary file 2 — Supplementary Material 2: Fig. S2 Screening Prognosis-Related Proteins with Proteomics. A Volcano plots of tumor focal vs para-cancerous tissues belonging to survival patients, tumor focal vs para-cancerous tissues belonging to dead patients and tumor focal tissues of survival patients vs dead patients. B KEGG pathway enrichment analysis of differential proteins. C Venn diagram of the intersection of three groups of differential proteins. D LASSO regression analysis for differential proteins yielded prognosis-associated proteins and their expression in different subgroups. E The changes of prognosis-associated proteins expression in different subgroups. F ROC curves of prognosis-associated proteins in different groups. G GSEA analysis of COX4I1 and UBL5-related signaling pathways. (*p<0.05; **p<0.01; ***p<0.001. C_D, Cancer tissues in death. N_D, Para-cancerous tissues in death. C_S, Cancer tissues in survival. N_S, Para-cancerous tissues in survival) [file 12943_2024_2039_MOESM2_ESM.pdf]

**A**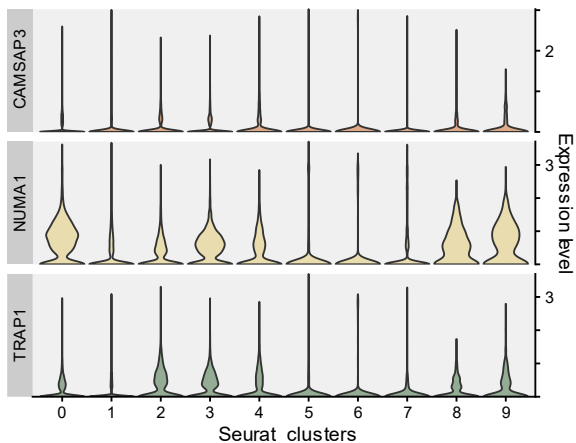**B**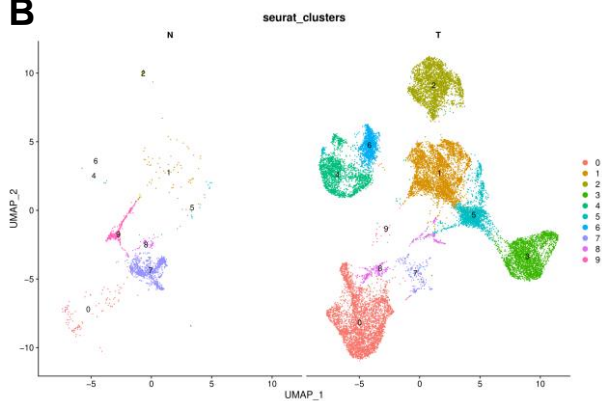**C**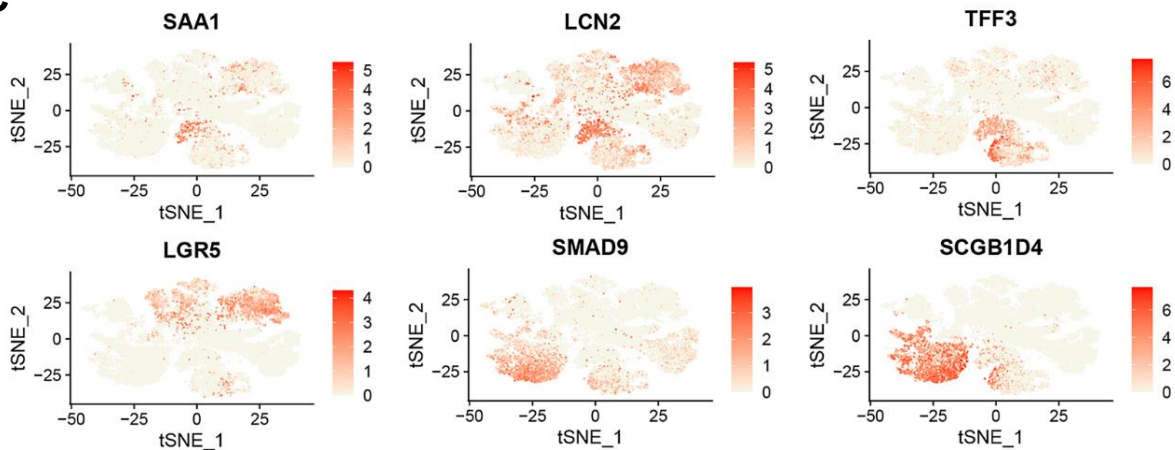**D**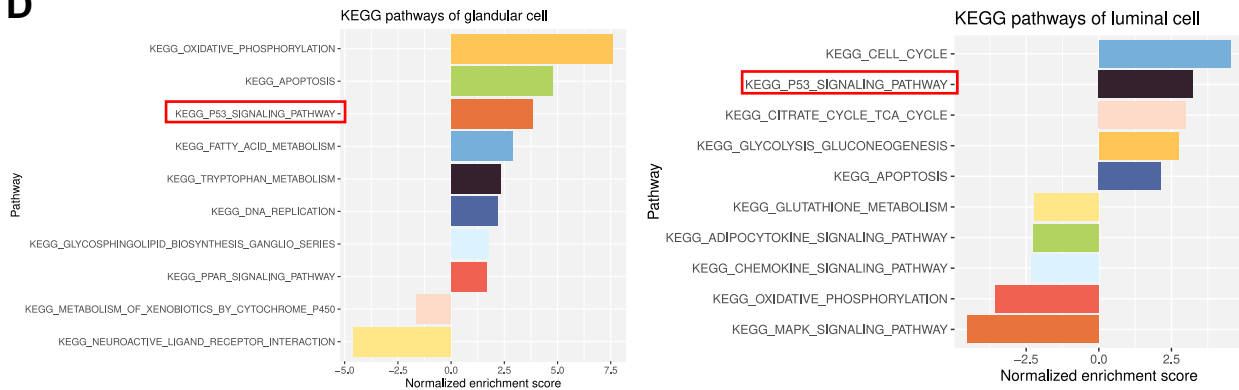

Supplement: Supplementary file 4 — Supplementary Material 4: Fig. S4 Expression of target protein in subpopulations. A Violin plot showed average gene expression of different clusters across all unciliated epithelial cells. B tSNE plot of the distribution of epithelial cells other than ciliated epithelial cells in tumor and normal samples. C t-SNE plots of canonical markers for epithelial subtypes. D GSEA analysis of glandular cells and luminal cells [file 12943_2024_2039_MOESM4_ESM.pdf]

**A**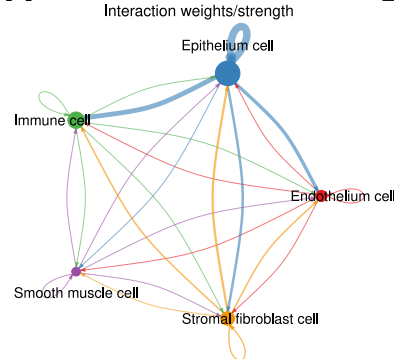**B**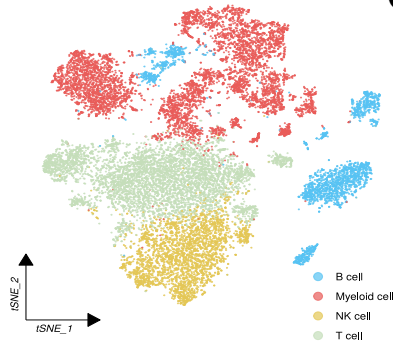**C**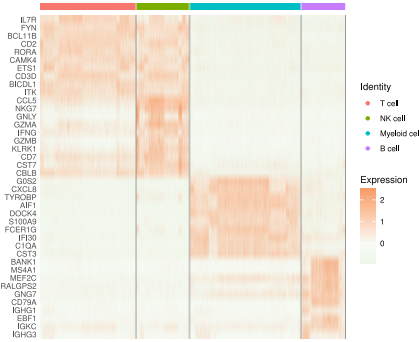**D**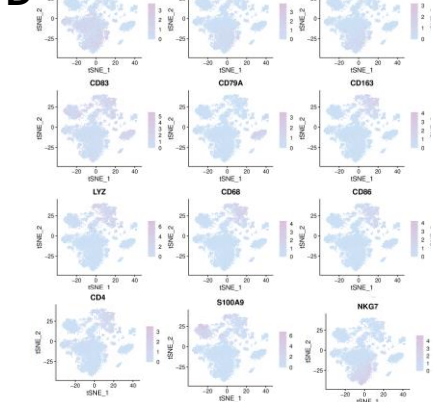**E**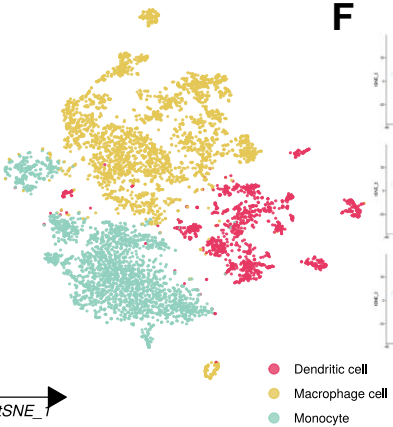**F**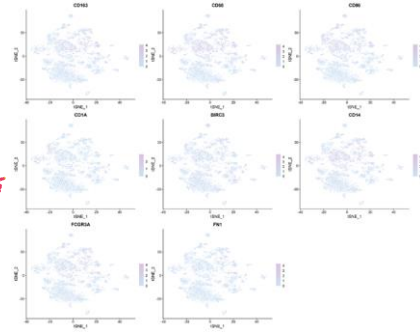**G**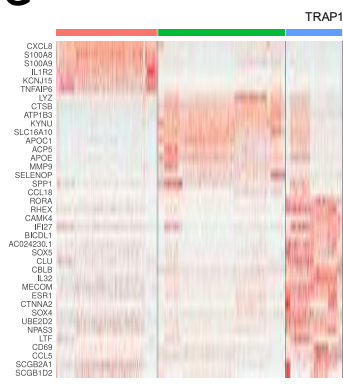**H**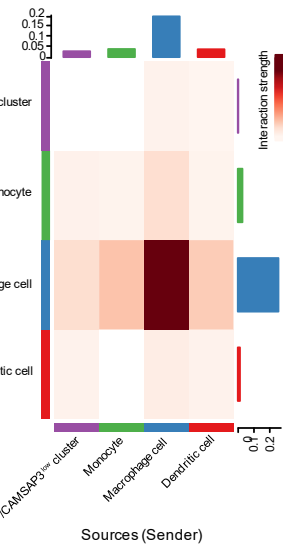**I**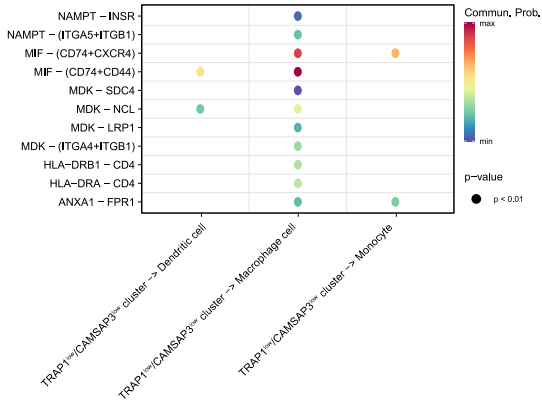

Supplement: Supplementary file 5 — Supplementary Material 5: Fig. S5 Myeloid cells, especially macrophages interacted closely with TRAP1low/CAMSAP3low cluster. A CellChat plot of intercellular communication between different cell types. B t-NSE plot of immune cells showed its major cell types. C Top genes in different cell subpopulations of immune cells. D Expression of cellular marker for each subpopulation of immune cells. E t-NSE diagram of myeloid cells. F Expression of cellular marker of different subpopulation in myeloid cells. G Top genes in different cell populations of myeloid cells. H CellChat plot of TRAP1low/CAMSAP3low cluster and myeloid cells. I Bubble chart showing the expression of receptor-ligand pairs in TRAP1low/CAMSAP3low cluster and myeloid cells [file 12943_2024_2039_MOESM5_ESM.pdf]

**A**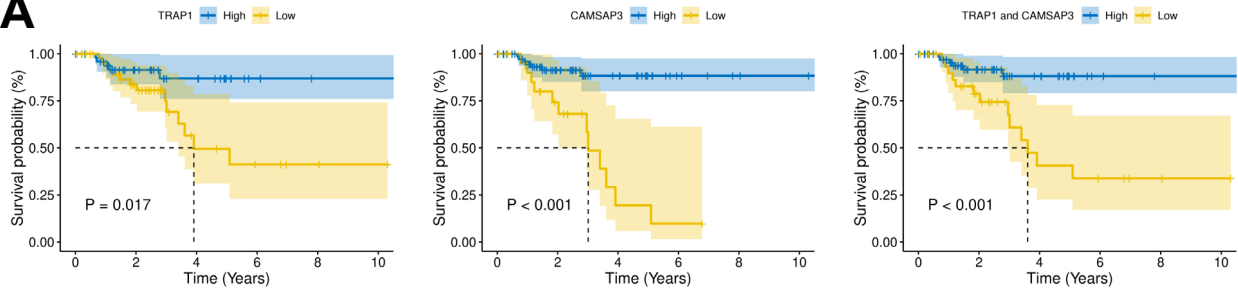**B**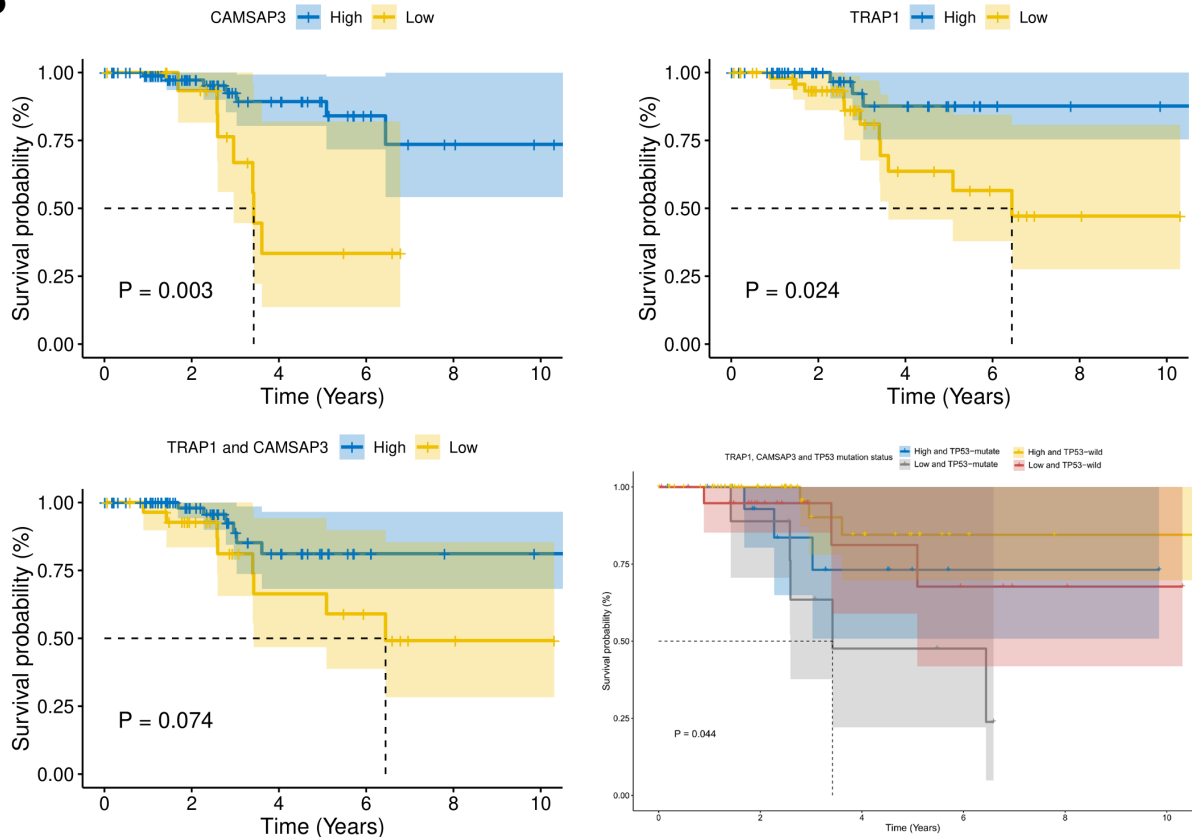

Supplement: Supplementary file 6 — Supplementary Material 6: Fig. S6 Survival curves of combined TRAP1, CAMSAP3 and TP53 mutation status across TCGA cohorts. A Combination with TRAP1 and CAMSAP3 predicted a disease outcome in EC patients without TP53 mutation. B Combination with TRAP1, CAMSAP3 and TP53 mutation status predicted a disease outcome in 106 stage I-II EC patients. Survival curves were visualized by Kaplan– Meier method. The endpoint was 10 years. The median was set as the cut-off value to stratify EC patients into low-expression and high-expression groups [file 12943_2024_2039_MOESM6_ESM.pdf]
