# Supplementary material for: Multi-omics profiling reveal cells with novel oncogenic cluster, TRAP1low/CAMSAP3low, emerge more aggressive behavior and poor-prognosis in early-stage endometrial cancer
Source: Mol Cancer. 2024 Jun 17;23:127. doi: 10.1186/s12943-024-02039-2 (PMC11181528; doi:10.1186/s12943-024-02039-2)
Supplement: Supplementary file 3 — Supplementary Material 3: Fig. S3 The derived cellular subpopulations of prognostic proteins were analyzed at the transcriptomic level. A t-SNE plots of cells from five patients (8 samples). Colors represented cell types. Cells were clustered into 5 cell types based on biological annotation. Each dot represents a single cell. B t-SNE plots of canonical markers for major cell types. C The expression levels of canonical marker genes for the above 5 cell types. Circle size represents the percentage of cells that expressed the gene, and colors represented the average expression value within a cluster. D The Venn plot showed the intersection between the prognostic proteins involved in the p53 signaling pathway identified by proteomics in tumor focal and para-cancerous tissues of survival patients and the differential genes of scRNA-seq. E Bar plot showing the expression of 3 genes in different cell types. F Bar chart showing the relative proportion of major cell types in each sample. G t-SNE plot showing clusters of epithelial. H Top genes in different cell populations of epithelial cells. I t-SNE plots of canonical markers for epithelial cells [file 12943_2024_2039_MOESM3_ESM.pdf]

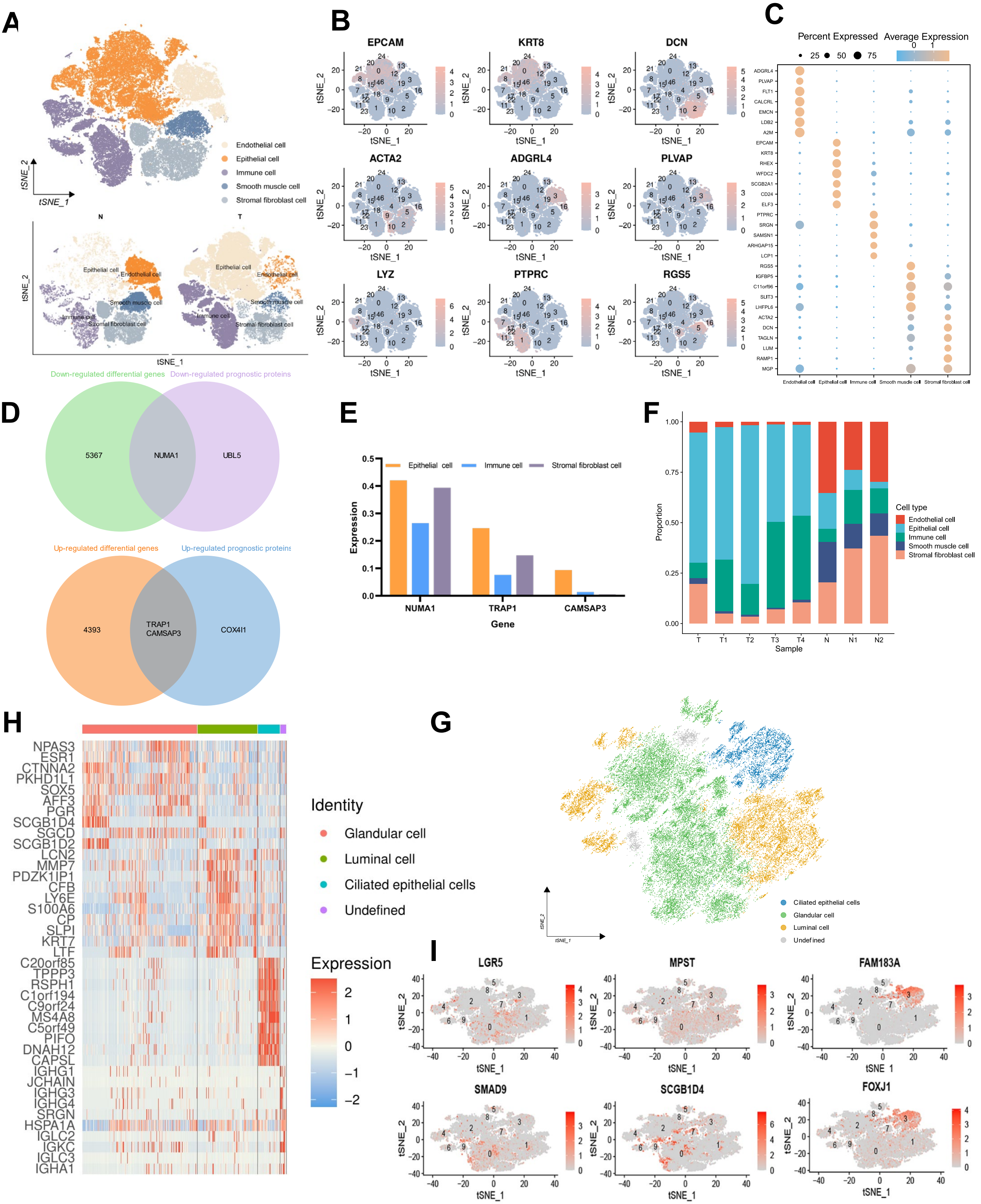
A
Endothelial cell
Epithelial cell
Immune cell
Smooth muscle cell
Stromal fibroblast cell
tSNE_1
tSNE_2
N
T
Epithelial cell
Endothelial cell
Smooth muscle cell
Immune cell
Stromal fibroblast cell
B
EPCAM
KRT8
DCN
ACTA2
ADGRL4
PLVAP
LYZ
PTPRC
RGS5
C
Percent Expressed
25
50
75
Average Expression
ADGRL4
PLVAP
FLT1
CALCRL
EMCN
LDB2
A2M
EPCAM
KRT8
RHEX
WFDC2
SCGB2A1
CD24
ELF3
PTPRC
SRGN
SAMSN1
ARHGAP15
LCP1
RGS5
IGFBP5
C11orf96
SLIT3
LHFPL6
ACTA2
DCN
TAGLN
LUM
RAMP1
MGP
Endothelial cell
Epithelial cell
Immune cell
Smooth muscle cell
Stromal fibroblast cell
D
Down-regulated differential genes
Down-regulated prognostic proteins
5367
NUMA1
UBL5
Up-regulated differential genes
Up-regulated prognostic proteins
4393
TRAP1
CAMSAP3
COX4I1
E
Epithelial cell
Immune cell
Stromal fibroblast cell
Expression
NUMA1
TRAP1
CAMSAP3
Gene
F
Proportion
Cell type
Endothelial cell
Epithelial cell
Immune cell
Smooth muscle cell
Stromal fibroblast cell
T
T1
T2
T3
T4
N
N1
N2
Sample
H
NPAS3
ESR1
CTNNA2
PKHD1L1
SOX5
AFF3
PGR
SCGB1D4
SGCD
SCGB1D2
LCN2
MMP7
PDZK1IP1
CFB
LY6E
S100A6
CP
SLPI
KRT7
LTF
C20orf85
TPPP3
RSPH1
C1orf194
C9orf24
MS4A8
C5orf49
PIFO
DNAH12
CAPSL
IGHG1
JCHAIN
IGHG3
IGHG4
SRGN
HSPA1A
IGLC2
IGKC
IGLC3
IGHA1
Identity
Glandular cell
Luminal cell
Ciliated epithelial cells
Undefined
Expression
G
Ciliated epithelial cells
Glandular cell
Luminal cell
Undefined
I
LGR5
MPST
FAM183A
SMAD9
SCGB1D4
FOXJ1
