# Supplementary material for: Multi-omics profiling reveal cells with novel oncogenic cluster, TRAP1low/CAMSAP3low, emerge more aggressive behavior and poor-prognosis in early-stage endometrial cancer
Source: Mol Cancer. 2024 Jun 17;23:127. doi: 10.1186/s12943-024-02039-2 (PMC11181528; doi:10.1186/s12943-024-02039-2)
Supplement: Supplementary file 7 — Supplementary Material 7: Table S1 Clinical characteristics of early-stage endometrioid endometrial cancer patients performed by proteomics. Table S2 Clinical characteristics of the IA stage (FIGO 2009) patients performed by scRNA-seq. Table S3 The mean expression levels (TPM) of prognostic genes in different clusters of unciliated epithelium. Table S4 The efficiency of different models on survival outcomes in EC patients from TCGA bank (N = 167). Supplementary methods and materials. Supplementary reference [file 12943_2024_2039_MOESM7_ESM.docx]

**Table S1 Clinical characteristics of early-stage endometrioid endometrial cancer patients performed by proteomics.**

|  | **Overall**  **(n=24)** | **Favourable prognosis**  **(n=15)** | **Poor prognosis**  **(n=9)** | **p-value** |  |
| --- | --- | --- | --- | --- | --- |
| **Age, years**  (median [IQR]) | 58.50  [55.75, 62.25] | 57.00  [53.00, 62.50] | 61.00  [56.00, 62.00] | 0.567 |  |
| **BMI, kg/m^2^**  (median [IQR]) | 23.69  [22.21, 25.90] | 23.24  [22.05, 25.71] | 24.92  [23.01, 25.87] | 0.676 |  |
| **Menopause** (n, %) |  |  |  | 1.000 |  |
| No | 4 (16.7) | 3 (20.0) | 1 (11.1) |  |  |
| Yes | 20 (83.3) | 12 (80.0) | 8 (88.9) |  |  |
| **Hypertension** (n, %) |  |  |  | 0.913 |  |
| No | 9 (37.5) | 5 (33.3) | 4 (44.4) |  |  |
| Yes | 15 (62.5) | 10 (66.7) | 5 (55.6) |  |  |
| **Diabetes** (n, %) |  |  |  | 1.000 |  |
| No | 15 (62.5) | 9 (60.0) | 6 (66.7) |  |  |
| Yes | 9 (37.5) | 6 (40.0) | 3 (33.3) |  |  |
| **FIGO stage (2009)** (n, %) |  |  |  | 0.595 |  |
| IA | 11 (45.8) | 8 (53.3) | 3 (33.3) |  |  |
| IB | 8 (33.3) | 4 (26.7) | 4 (44.4) |  |  |
| II | 5 (20.9) | 3 (20.0) | 2 (22.2) |  |  |
| **Pathological grade** (n, %) |  |  |  | 0.004 |  |
| Grade I | 13 (54.2) | 11 (73.3) | 2 (22.2) |  |  |
| Grade II | 6 (25.0) | 4 (26.7) | 2 (22.2) |  |  |
| Grade III | 5 (20.8) | 0 (0.0) | 5 (55.6) |  |  |
| **Average survival time, months** | 86.125  [67.788, 104.462] |  |  |  |  |
| **Follow-up time, months**  (Min, Max, Median) | 5, 120, 108 | 84, 120, 108 | 5, 84, 27 |  |  |
| **ER** (n, %) | |  |  |  | 0.224 |
| -~+ | 6 (25.0) | 2 (13.3) | 4 (44.4) |  |  |
| ++~+++ | 18 (75.0) | 13 (86.7) | 5 (55.6) |  |  |
| **PR** (n, %) |  |  |  | 0.417 |  |
| -~+ | 7 (29.2) | 3 (20.0) | 4 (44.4) |  |  |
| ++~+++ | 17 (70.8) | 12 (80.0) | 5 (55.6) |  |  |
| **Ki67** (n, %) |  |  |  | 1.000 |  |
| <30% | 3 (12.5) | 2 (13.3) | 1 (11.1) |  |  |
| ≥30% | 21 (87.5) | 13 (86.7) | 8 (88.9) |  |  |
| **Vimentin** (n, %) |  |  |  | 0.472 |  |
| -~+ | 11 (45.8) | 7 (46.7) | 4 (44.4) |  |  |
| ++~+++ | 11 (45.8) | 6 (40.0) | 5 (55.6) |  |  |
| Unknown | 2 (8.3) | 2 (13.3) | 0 (0.0) |  |  |
| **TP53** (n, %) |  |  |  | 0.006 |  |
| Wild | 19 (79.2) | 15 (100.0) | 4 (44.4) |  |  |
| Mutate | 5 (20.8) | 0 (0.0) | 5 (55.6) |  |  |
| **MMR** (n, %) |  |  |  | 1.000 |  |
| MMRd | 17 (70.8) | 11 (73.3) | 6 (66.7) |  |  |
| MMRp | 7 (29.2) | 4 (26.7) | 3 (33.3) |  |  |

**Notes:** For continuous nonnormal variables, it showed median and IQR, and p-value was from nonparametric tests. For categorical variables, it showed the percentage that each variable accounted for. BMI, body mass index. ER, estrogen receptor. PR, progesterone receptor. MMRd, mismatch repair deficiency. MMRp, mismatch repair proficiency.

**Table S2 Clinical characteristics of the ⅠA stage（2009）patients performed by scRNA-seq.**

|  | **Endometrioid endometrial cancer (n=5)** | |
| --- | --- | --- |
| **Age, years**  (median [IQR]) | 53.00  [49.00, 57.00] |  |
| **BMI, kg/m^2^**  (median [IQR]) | 26.02  [22.77, 26.04] |  |
| **Menopause** (n, %) |  |  |
| No | 2 (40.0) |  |
| Yes | 3 (60.0) |  |
| **Hypertension** (n, %) |  |  |
| No | 3 (60.0) |  |
| Yes | 2 (40.0) |  |
| **Diabetes** (n, %) |  |  |
| No | 5 (100.0) |  |
| Yes | / |  |
| **Pathological grade** (n, %) |  |  |
| Grade I | 3 (60.0) |  |
| Grade II | 1 (20.0) |  |
| Grade III | 1 (20.0) |  |
| **ER** (n, %) |  |  |
| -~+ | 2 (40.0) |  |
| ++~+++ | 3 (60.0) |  |
| **PR** (n, %) |  |  |
| -~+ | 3 (60.0) |  |
| ++~+++ | 2 (40.0) |  |
| **Ki67** (n, %) |  |  |
| ≥30%  ＜30% | 5 (100.0)  / |  |
| **Vimentin** (n, %) |  |  |
| -~+ | 3 (60.0) |  |
| ++~+++ | 2 (40.0) |  |
| **TP53** (n, %) |  |  |
| Wild | 5 (100.0) |  |
| Mutation | / |  |
| **MMR** (n, %) |  |  |
| MMRd | 4 (80.0) |  |
| MMRp | 1 (20.0) |  |

**Notes:** For continuous nonnormal variables, it showed median and IQR, and p-value was from nonparametric tests. For categorical variables, it showed the percentage that each variable accounts for. BMI, body mass index. ER, estrogen receptor. PR, progesterone receptor. MMRd, mismatch repair deficiency. MMRp, mismatch repair proficiency.

**Table S3 The mean expression levels (TPM) of prognostic genes in different clusters of unciliated epithelium.**

| **Cluster** | **Num. of cells** | ***CAMSAP3*** | ***NUMA1*** | ***TRAP1*** | **Expression level** |
| --- | --- | --- | --- | --- | --- |
| **0** | 4118 | 0.112119527 | 0.716973644 | 0.202207225 | High |
| **1** | 4001 | 0.067060445 | 0.340807387 | 0.216029197 | High |
| **2** | 2890 | 0.104658737 | 0.33881228 | 0.364490961 | High |
| **3** | 2666 | 0.11778535 | 0.515986776 | 0.336401044 | High |
| **4** | 2094 | 0.126537558 | 0.398889698 | 0.284300215 | High |
| **5** | 1836 | 0.039498042 | 0.204123283 | 0.153107086 | Low |
| **6** | 1245 | 0.039543159 | 0.137856174 | 0.09589564 | Low |
| **7** | 1122 | 0.01727068 | 0.368373059 | 0.118272246 | Low |
| **8** | 517 | 0.068019633 | 0.53298313 | 0.207547883 | High |
| **9** | 339 | 0.098214813 | 0.622363084 | 0.257347278 | High |
| **Median** |  | 0.083117223 | 0.383631379 | 0.21178854 |  |

**Notes:** The gene expression of each cell in each cluster was extracted and then averaged. The respective median for each gene was set as the threshold value to stratify cluster into two groups. When the expression level was less than the median of all three genes, it was defined as “Low”, otherwise it was defined as “High”. *CAMSAP3,* calmodulin-regulated spectrin-associated protein 3. *NUMA1,* nuclear mitotic apparatus protein 1. *TRAP1,* tumor necrosis factor type 1 receptor-associated protein.

**Table S4 The efficiency of different Models on survival outcomes in EC patients from TCGA bank (n=167).**

|  | ***TP53*** | ***TRAP1*** | ***CAMSAP3*** | **AUC**  **(95% CI)** | **SEN%**  **(95% CI)** | **SPE%**  **(95% CI)** | **PPV%**  **(95% CI)** | **NPV%**  **(95% CI)** | **p-value** |
| --- | --- | --- | --- | --- | --- | --- | --- | --- | --- |
| **Model1** | √ |  |  | 0.72  (0.63-0.80) | 66.7  (54.4-78.9) | 76.4  (68.4-84.3) | 59.4  (47.3-71.4) | 81.6  (74.1-89.0) |  |
| **Model2** |  | √ |  | 0.75  (0.67-0.83) | 73.7  (62.3-85.1) | 76.4  (68.4-84.3) | 61.8  (50.2-73.3) | 84.8  (77.8-91.9) | 0.479 |
| **Model3** |  |  | √ | 0.65  (0.56-0.73) | 70.2  (58.3-82.1) | 59.1  (49.9-68.3) | 47.1  (36.4-57.7) | 79.3  (70.5-88.0) | 0.182 |
| **Model4** | √ | √ |  | 0.83  (0.76-0.90) | 73.7  (62.3-85.1) | 76.4  (68.4-84.3) | 61.8  (50.2-73.3) | 84.8  (77.8-91.9) | 0.002 |
| **Model5** | √ |  | √ | 0.77  (0.70-0.84) | 66.7  (54.4-78.9) | 76.4  (68.4-84.3) | 59.4  (47.3-71.4) | 81.6  (74.1-89.0) | 0.006 |
| **Model6** | √ | √ | √ | 0.84  (0.78-0.91) | 70.2  (58.3-82.1) | 85.5  (78.9-92.0) | 71.4  (59.6-83.3) | 84.7  (78.0-91.4) | <0.001 |

**Notes:** All p-values were derived by comparing other models with model1 according to the AUC. *CAMSAP3,* calmodulin-regulated spectrin-associated protein 3. *NUMA1,* nuclear mitotic apparatus protein 1. *TRAP1,* tumor necrosis factor type 1 receptor-associated protein. AUC, area under the receiver operator characteristic curve; SEN, sensitivity; SPE, specificity; PPV, positive predictive value; NPV, negative predictive value; CI, Confidence interval.

**Supplementary materials and methods**

**Specimens and clinical data**

In this study, patients with FIGO stage I-II disease were selected for analysis, and the histological type was endometrioid endometrial cancer (EEC). All samples involved in this study were obtained from Fujian Maternity and Child Health Hospital, Fujian Medical University, and received approval from the ethical committee. The samples included 24 proteomics FFPE samples, 8 fresh tissue samples used for scRNA-seq and 1 FFPE sample used for spatial transcriptomics. Normal endometrial tissues were paired with para-cancerous tissues from patients with EC, and no malignant lesions were confirmed according to pathological sections.

The proteomics analysis and immunohistochemistry reviewed patients who underwent surgery for endometrioid endometrial cancer (EEC) in our hospital between May 2013 and May 2023. Inclusion criteria were as follows: patients with complete clinical information, well-preserved pathological wax blocks, and no history of hormone therapy within six months prior to surgery, as well as no other concurrent tumors or infectious diseases. After matching the patients based on follow-up time, age, BMI, and FIGO stage, 15 surviving patients and 9 dead patients were selected for further research. Combined with clinical information, patients who died within 10 years as the poor prognosis, and those who survived within 10 years as the favorable prognosis.

Each patient signed an informed consent and an authorization form before the experiment.

**Proteomic sample preprocessing**

FFPE biobank specimens (5 serial sections, 10 mM thick) were first deparaffinized by two washes in 1 mL of xylene (1 hour at 37°C each) followed by two washes in 1 mL of absolute ethanol. Ethanol was removed completely, and the sections were air-dried. The samples were dewaxed with xylene. After that, four volumes of lysis buffer (1% SDS, 0.1 M Tris/HCl, pH 7.5, 1% protease inhibitor cocktail) were added to the sample, which was subsequently incubated at 95°C for 120 min at 600 rpm on a heater and cooled to RT, followed by sonication for three minutes on ice using a high-intensity ultrasonic processor (Scientz). After centrifugation for 10 min at 16 000 × g at 4°C, proteins in the supernatant were precipitated with 4-fold excess v/v of -20°C acetone overnight. The pellet was isolated by centrifugation at 10 000 × g at 4°C for 10 min, washed with 80–20°C acetone, resuspended in 100 μL of 0.2 M TEAB and ultrasonically dispersed. Trypsin was added at a 1:50 trypsin-to-protein mass ratio for the first digestion overnight. The sample was reduced with 5 mM dithiothreitol for 60 min at 37°C and alkylated with 11 mM iodoacetamide for 45 min at room temperature in darkness. Finally, the peptides were desalted on a Strata X SPE column.

**LC–MS/MS analysis**

Tryptic peptides dissolved in solvent A (0.1% FA) were directly loaded onto an Evotip following the manufacturer’s protocol. Peptides were separated with the preset 60-Samples per Day method on the EvoSep, with the mobile phase consisting of solvent A and solvent B (0.1% FA in ACN). The peptides were subjected to capillary source followed by mass spectrometry on a timsTOF Pro mass spectrometer. The electrospray voltage applied was 1.6 kV. Precursors and fragments were analyzed with a TOF detector. The timsTOF Pro was operated in data independent parallel accumulation serial fragmentation (dia-PASEF) mode. The full MS scan range was set as 100-1700 m/z, and 10 PASEF scans were acquired per cycle. The MS/MS isolation window was set at 35 m/z in the range of 400-1200 m/z.

**Analysis of MS/MS data**

The DIA data were processed using the DIA-NN search engine (v.1.8). Tandem mass spectra were searched against Homo_sapiens_9606_SP_20230103.fasta (20389 entries) concatenated with a reverse decoy database. Trypsin/P was specified as a cleavage enzyme allowing up to 1 missing cleavage. Excision of N-term Met and carbamidomethyl on Cys were specified as fixed modifications. The FDR was adjusted to < 1%.

**Bioinformatics analysis**

Proteins with less than 50% missing values were selected, and missing values were then imputed based on a normal distribution. Significance was tested using an unpaired two-sided Student’s t test, and a p value < 0.05 and a fold change >1.2 or <0.833 were considered significant. Kyoto Encyclopedia of Genes and Genomes (KEGG) was used for pathway enrichment analysis, and the results were clustered together using the R package pheatmap and plotted as a heatmap. The cv.glmnet function in the glmnet package of R was used for LASSO regression. The ROC curves were plotted using the pROC package. For the 13 proteins most related to prognosis screened, the relative quantitative Pearson correlation between them and all other proteins was calculated. The correlation was used as the rank of GSEA for enrichment analysis, and the annotation library was KEGG. The top (p value<0.05 and NES >1 or < -1) pathways positively or negatively correlated with each marker protein were screened, and the combined enrichment map was drawn in R.

**Single-cell collection and library construction**

The tumor tissues and paired paracancerous tissues were cut into small pieces of approximately 1-3 mm³. Cell capture and cDNA synthesis were performed using a single-cell 3' Library and Gel Bead Kit V3.1 (10x Genomics, 1000121) and a Chromium Single-Cell G Chip Kit (10x Genomics, 1000120). The cell suspension (300-600 living cells per microliter determined by Count Star) was loaded onto a chromium single-cell controller (10x Genomics) to generate single-cell gel beads in the emulsion according to the manufacturer’s protocol. ScRNA-seq library preparation According to the manufacturer’s instructions, single-cell RNA-seq libraries were constructed using the Single-cell 3’ Library and Gel Bead Kit V3.1. The libraries were finally sequenced using an Illumina NovaSeq 6000 sequencer with a sequencing depth of at least 100,000 reads per cell via a paired-end 150 bp (PE150) strategy (performed by CapitalBi Reading Technology, Beijing).

**Quality control**

The raw EC cell data were processed through Cell Ranger (10x Genomics). A raw unique molecular identifier (UMI) count matrix was generated after Cell Ranger processing, and the matrix was subsequently converted into a Seurat object and analyzed by the Seurat package in R. Cells whose gene number was less than 200, whose gene number ranked in the top 1%, or whose mitochondrial gene ratio was more than 25% were regarded as abnormal and filtered out.

**UMAP/t-SNE Clustering Analysis.**

For clustering, we first ran PCA and selected the top 30 PCAs to find clusters. The Seurat package was used for clustering and t-distributed stochastic neighbor embedding (t-SNE). Clustering analysis was performed with FindClusters, and the resolution was set to 0.1-0.6. Run-tSNE was used to visualize the samples. Seurat function FindMarkers and FindAllMarkers were used to identify DEGs with the Wilcoxon test(min.pct = 0.1, test.use = ‘BH’, return.thresh = 0.25).

**GSEA**

GSEA implemented with the fGSEA package was used for cell subcluster enrichment analysis. The KEGG pathway gene sets were exported by using the msigdbr package (version 7.5.1) and the gene set files of the website (version 7.4). The activities of pathways between cells in different groups were scored with the limma package. The top (p value<0.05 and NES >1 or < -1) pathways positively or negatively correlated with the different cell subpopulations were screened, and the chosen pathways were visualized with the ggplot2 package.

**Intercellular communication/interaction**

The CellChat package (version 1.4.0) and CellChat database were used to analyze and infer cell‒cell communication[1]. All the statistical analyses were performed using R (version 4.3.2). All figures were plotted by using R. p values < 0.05 or p values <0.05 were considered to indicate statistical significance.

**Immunohistochemical staining**

For the immunohistochemical staining experiment, 24 pairs of EC specimens were obtained from patients who received surgical treatment at Fujian Maternity and Child Health Hospital. The tissue sections were dehydrated, repaired with citrate antigen, and blocked with 5% goat serum for 15 min at room temperature. The tissue sections were stained with diluted primary anti-TRAP1 (ABclonal, A4416, 1:200), anti-NUMA1 (ABclonal, A2748, 1:100) and anti-CAMSAP3 (SAB, #47694, 1:800) antibodies at 4 °C overnight. Subsequently, the tissue sections were incubated with DAB, counterstained with hematoxylin, and dehydrated with ethanol. Two independent pathologists examined and scored all sections in a double-blind manner. Immunohistochemical scoring was performed using a quantitative method. Three low-power visual fields (× 100) were randomly selected under the microscope, and the corresponding scores of strong staining, moderate staining, weak staining, and negative staining were 3, 2, 1 and 0, respectively, according to the positive staining intensity. The proportion of stained cells was divided into 76% ~ 100%, 51% ~ 75%, 26% ~ 50%, and 0 ~ 25%, and the corresponding scores were 4, 3, 2 and 1 points, respectively. The intensity and percentage of positively stained cells were multiplied to calculate the immunohistochemical score of the sample.

**TCGA database analysis**

The mRNA sequencing data from 589 patients with UCEC were downloaded from TCGA. The patients were divided into the early group (stage I-II, n=395), advanced group (stage III-IV, n=159) and normal population (n=35) according to their FIGO stage for difference analysis. The median was set as the cutoff value to stratify EC patients into low-risk and high-risk groups. Kaplan-Meier (K-M) analysis was performed to calculate the difference in DSS between the low-risk and high-risk groups. Data from EC patients with *TP53* mutation status were obtained from cBioPortal[2,3], and 57 patients who died due to EC and 110 surviving patients were screened for receiver operating characteristic (ROC) analysis by propensity score matching. All the data were visualized with the ggplot2 package.

**Statistical analysis**

The experiment was not randomized. Researchers were not blinded to the methods used in the experiments or in the evaluation of the results. Data collection and analysis are not limited by experimental conditions. Statistical analysis was performed using R 4.3.2 and GraphPad Prism 9.5 software. The Wilcoxon rank sum test was used to compare variables between treatment conditions and responses. For the expression of genes in different EC staging samples, the Kruskal‒Wallis test with Dunn’s correction was used for statistical analysis, and p < 0.05 was considered to indicate statistical significance. DSSs were calculated using K–M analysis and compared with the log-rank test. For the comparison of more than two groups, analysis of variance was used for IHC. P values less than 0.05 were considered to indicate statistical significance.

**Supplementary reference**

1. Jin S, Guerrero-Juarez CF, Zhang L, Chang I, Ramos R, Kuan C-H, et al. Inference and analysis of cell-cell communication using CellChat. Nat Commun. 2021;12:1088.

2. Cerami E, Gao J, Dogrusoz U, Gross BE, Sumer SO, Aksoy BA, et al. The cBio cancer genomics portal: an open platform for exploring multidimensional cancer genomics data. Cancer Discov. 2012;2:401–4.

3. Hoadley KA, Yau C, Hinoue T, Wolf DM, Lazar AJ, Drill E, et al. Cell-of-Origin Patterns Dominate the Molecular Classification of 10,000 Tumors from 33 Types of Cancer. Cell. 2018;173:291-304.e6.
